# Case Report: Effectiveness of deucravacitinib in chronic recurrent multifocal osteomyelitis and concomitant psoriasis

**DOI:** 10.3389/fimmu.2025.1480810

**Published:** 2025-05-22

**Authors:** Caroline Glatzel, Patrick-Pascal Strunz, Matthias Goebeler, Annette Holl-Wieden, Marc Schmalzing, Astrid Schmieder

**Affiliations:** ^1^ Department of Dermatology, Venereology and Allergology, University Hospital Würzburg, Würzburg, Germany; ^2^ Department of Internal Medicine II, Rheumatology/Clinical Immunology, University Hospital of Würzburg, Würzburg, Germany; ^3^ Children’s Hospital, Section of Pediatric Rheumatology, University of Würzburg, Würzburg, Germany

**Keywords:** deucravacitinib, CRMO, CNO, paradox psoriasis, JAK-inhibitor

## Abstract

We present the case of a 22-year-old male who was first diagnosed with chronic recurrent multifocal osteomyelitis (CRMO) at the age of 16 years. During treatment with the tumor necrosis factor (TNF)-alpha inhibitor adalimumab, he developed severe palmoplantar psoriasis that affected his quality of life and necessitated discontinuation of the drug despite its efficacy against CRMO. Subsequent treatment with the interleukin (IL)-17a inhibitor secukinumab did not improve the psoriasis and led to a recurrence of the osteomyelitis. Therefore, the TYK-2 inhibitor deucravacitinib, approved in 2023 for the treatment of moderate to severe plaque psoriasis, was initiated. This resulted in clinical and morphological complete and sustained remission of CRMO within 12 weeks and a significant improvement of the psoriasis after six months of treatment with no occurrence of severe adverse events. To our knowledge, this is the first case report demonstrating complete and safe remission of CRMO associated with palmoplantar psoriasis treated with the TYK2 inhibitor deucravacitinib.

## 1 Introduction

Chronic recurrent multifocal osteomyelitis (CRMO) is a rare, chronic inflammatory bone disease that can lead to bone destruction if left untreated. It occurs primarily in children between the age of 3 and 17 (1) and may be associated with chronic inflammatory bowel disease, severe acne, ankylosing spondylitis, and psoriasis, including palmoplantar pustulosis (2). Typically, the osteomyelitis affects the metaphyses of the long bones, especially those of the femur and tibia, but other bones such as the pelvis, spine, clavicle, and mandible may be involved too. Because of the multifocality of bone lesions, experts in the field emphasize the importance of using whole-body MRI for diagnosis and follow-up of the disease to prevent long-term sequelae such as vertebral collapse (1, 3).

The exact pathophysiology of CRMO is still unclear. While bacteria are not the cause of CRMO, a cytokine imbalance has been described in the literature with decreased concentrations of anti-inflammatory cytokines such as interleukin (IL)-10 and increased pro-inflammatory cytokines such as IL-1β, IL-6 and tumor necrosis factor alpha (TNF-α) (4–6). Single gene defects have been identified in a subset of patients who are affected by CRMO such as LPIN2 gene mutations (7).

Treatment options for CRMO include non-steroidal anti-inflammatory drugs, methotrexate, sulfasalazine, bisphosphonates, short courses of glucocorticoids, and TNF-α inhibitors(i) (8). Other biologics such as IL-1i, IL-23i, IL-6i, and IL-17i are discussed as potential new treatment options (9, 10). Regarding small molecular weight inhibitors, evidence currently exists only for the Janus kinase inhibitor (JAKi) tofacitinib in the treatment of CRMO (11), and to the best of our knowledge, no other JAK inhibitors have been described as effective in treating this form of osteomyelitis.

## 2 Case presentation

**Figure 1:**
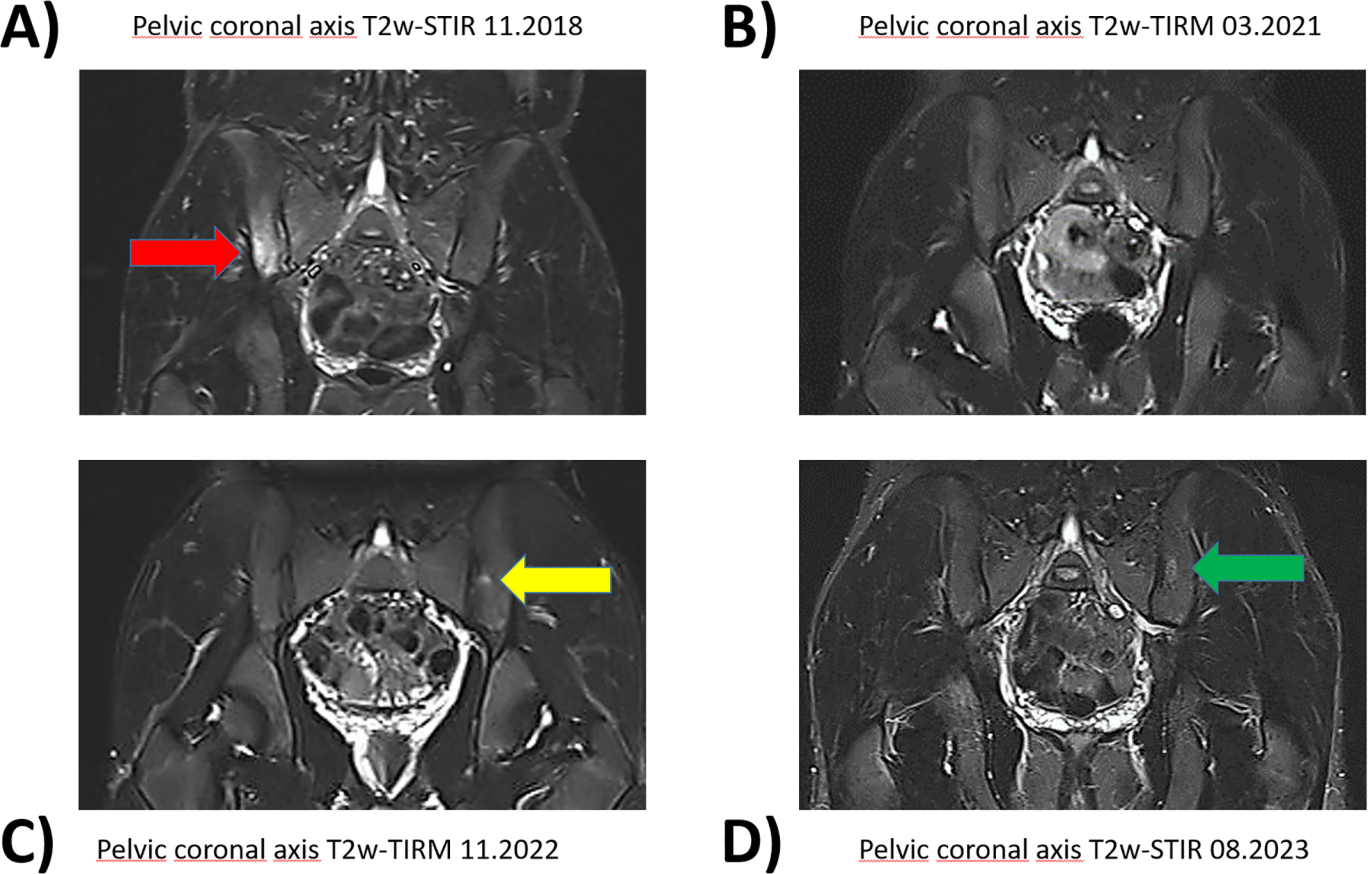
Pelvic magnetic resonance imaging with fat suppressed sequences. Corresponding pelvic coronary planes in MRI imaging during the course of the disease between 11.2018 and 08.2023. The fat suppressed sequences T2-weighted short-tau inversion recovery (T2w-STIR) and T2-weighted turbo inversion recovery magnitude (T2w-TIRM) were used for image acquire. **(A)** Hyperintense edema signal representing osteitis was found in the right iliac bone and adjacent lateral mass in 11.2018(red arrow). **(B)** Adalimumab treatment led to a good response with regression of edema. **(C)** New lesion on the left side after discontinuation of adalimumab 05.2022 (yellow arrow). **(D)** Persisting inflammation despite initiation of secukinumab in 11.2022 (green arrow).

A 16-year-old male patient first presented to the pediatric rheumatology clinic in November 2018 with gradually increasing typically inflammatory back pain in the area of the sacroiliac joints, which had been treated with naproxen and metamizol for 6 weeks. Since summer 2018, he has also suffered from severe acne conglobata on his back, décolletage, and face. He has been treated with oral isotretinoin 30 mg once daily (according to 0.5 mg/kg body weight). His family history is negative for rheumatologic disease, psoriasis, or chronic inflammatory bowel disease. Whole-body MRI (see **Figure 1**) showed a significant edema signal compatible with osteitis with a punctum maximum in the right ilium (**Figure 1**). At this time, inflammatory markers were moderately elevated (c-reactive protein [CRP] 1.64 mg/dl (0-0.5), erythrocyte sedimentation rate [ESR] 30 mm/1h).

Based on these findings, the pediatric rheumatologist diagnosed chronic nonbacterial osteomyelitis (CRMO) with acne conglobata and added sulfasalazine to the treatment regimen. The inflammatory levels normalized over the following weeks. However, the patient reported renewed severe inflammatory back pain in the left pelvis with radiation in his thighs, and occasionally in the thoracic spine. Sulfasalazine, which the patient had been taking for 4 months, was discontinued in March 2019 and treatment with the TNF-i adalimumab 40 mg s.c. every 2 weeks was initiated.

Eight weeks after starting adalimumab, the inflammatory back pain had improved significantly and the acne conglobata had almost resolved. Naproxen and isotretinoin were discontinued. Follow-up whole-body MRIs in September 2019 showed only residual edema signals (iliac bone and lateral mass of the sacral bone) and significantly reduced remnants of chronic non-bacterial osteomyelitis (CRMO).

Two and a half years after starting adalimumab, in September 2021, the patient suddenly developed extensive erythematous scaly plaques and rhagades on the palms and lower legs, suspected to be a paradoxical psoriasis due to adalimumab therapy. The patient’s quality of life was severely impaired by the pain from these lesions, especially on the palms of his hands, which was particularly challenging given his work as a craftsman (Dermatology Life Quality Index: 22, May 2022). As a result of the pronounced skin symptoms, adalimumab was discontinued after 38 months in May 2022. The dermatology team was consulted, and acitretin 30 mg capsules were initiated in September 2022. However, this treatment only provided short-term improvement of the palmoplantar psoriasis. Clearly infiltrated erythrosquamous plaques developed on the lower legs, palms of the hands, and, newly, on the feet, accompanied by nail changes typical of psoriasis. Consequently, acitretin was discontinued.

Two months after discontinuing adalimumab, in July 2022, the patient reported recurrent pain in the sacroiliac joint area for the first time. However, a whole-body MRI showed no active bone lesions. Since the skin symptoms had become the primary issue, pediatric rheumatologists initiated secukinumab treatment in November 2022 after consulting with the dermatologists, starting with 150 mg and then increasing to 300 mg subcutaneously every four weeks. The palmoplantar psoriasis persisted almost unchanged, and abscesses and infections frequently developed on the lower legs, some of which required surgical intervention.

However, nine months after switching the systemic therapy to the IL-17i secukinumab, the whole-body MRI (August 2023) showed a recurrence with new lesions suspicious for CRMO in the proximal parts of both the fibula and tibia, as well as in the previously known lesions in the sacroiliac joints.

**Figure 2:**
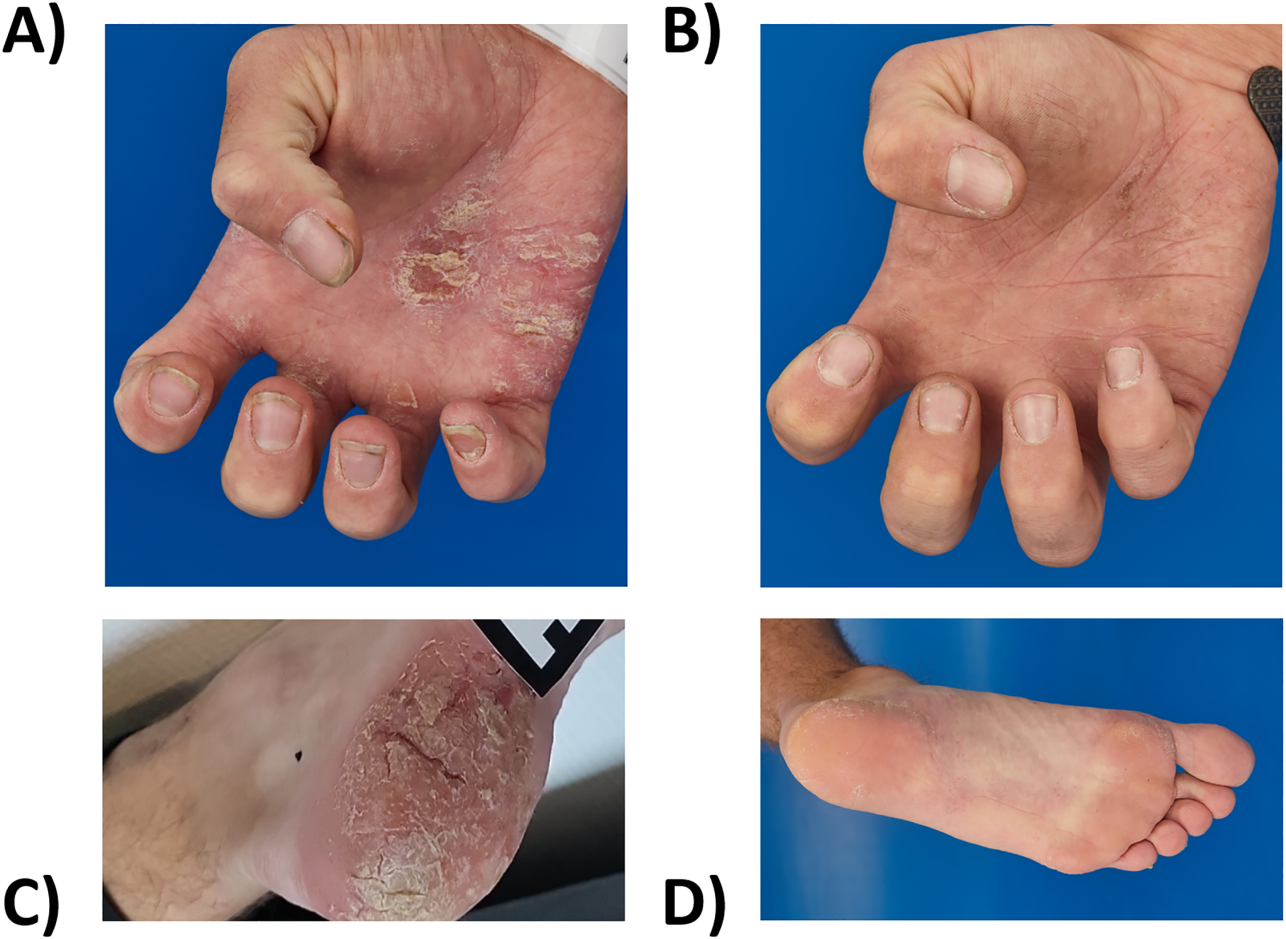
Extensive erythematous scaly plaques and rhagades on the palms and soles **(A, C)** before (October 2023) and **(B, D)** after eight months of deucravacitinib treatment (June 2024).

**Figure 3:**
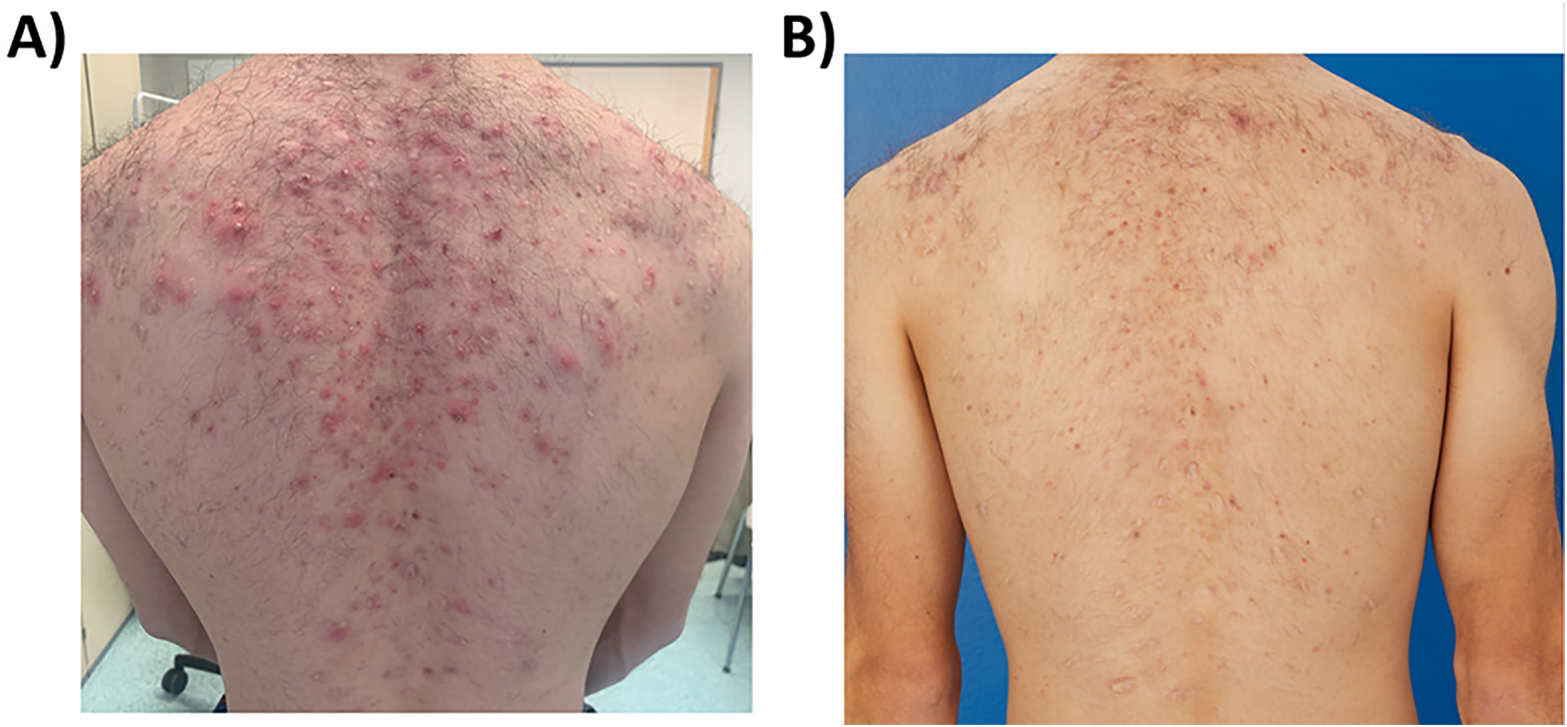
Acne-like eruptions developed on the back during deucravacitinib treatment **(A)** (march 2024), which rapidly improved with topical adapalene and benzoyl peroxide and systemic doxycycline 50 mg daily **(B)** (June 2024).

**Figure 4:**
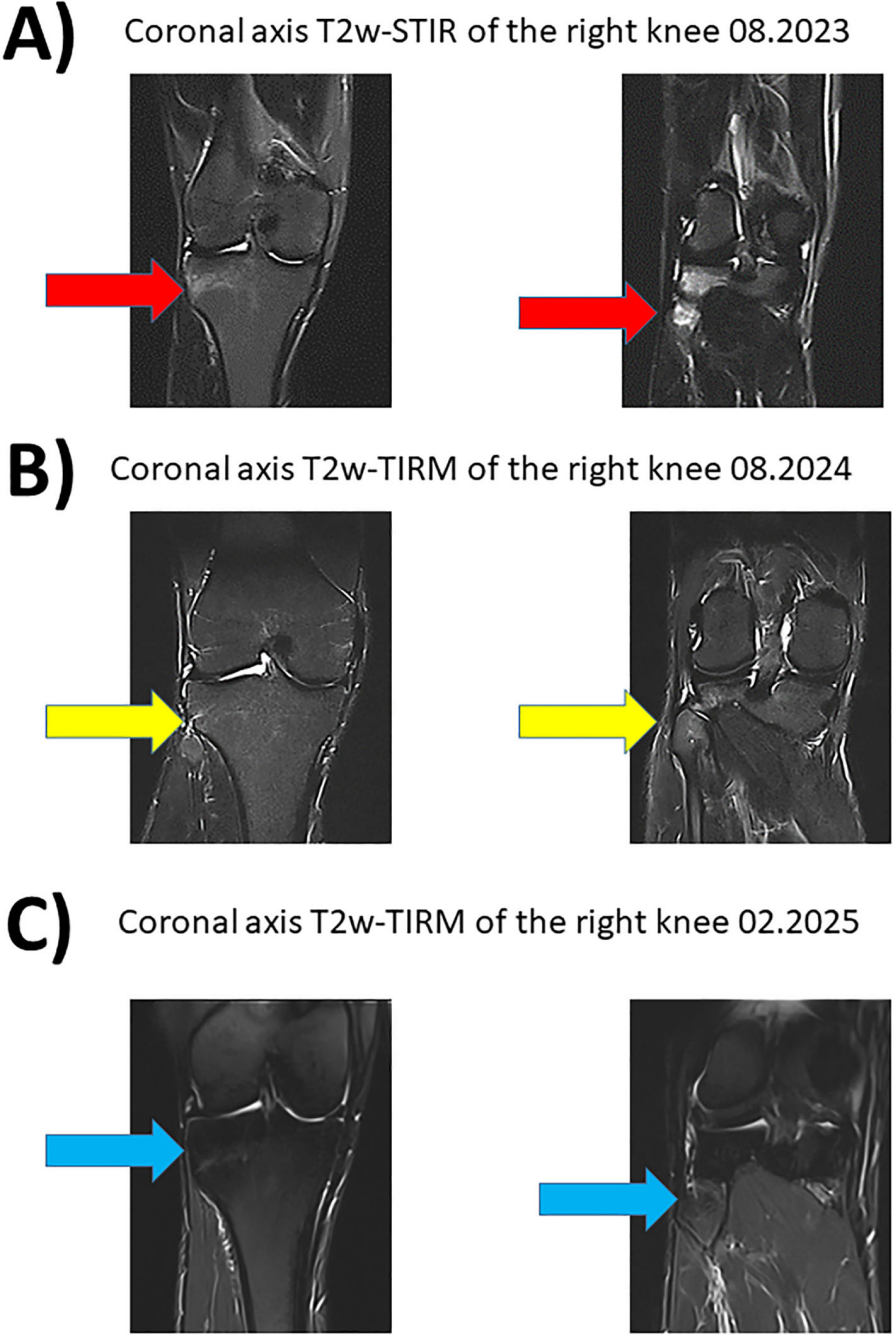
Magnetic resonance imaging of the right knee with fat suppressed sequences. Corresponding coronary planes of the right knee in MRI imaging during the course of the disease between 08.2023 and 08.2024. The fat suppressed sequences T2-weighted short-tau inversion recovery (T2w-STIR) and T2-weighted turbo inversion recovery magnitude (T2w-TIRM) were used for image acquise. **(A)** Hyperintense edema signal representing new osteitis under IL-17i was found in the right tibia and fibula in 11.2024 (red arrow). **(B)** Treatment with deucravacitinib led to good response with regression of the hyperintense lesions (yellow arrow). **(C)** Sustained and stable remission for 15 months under treatment with deucravacitinib (blue arrow).

**Figure 5:**
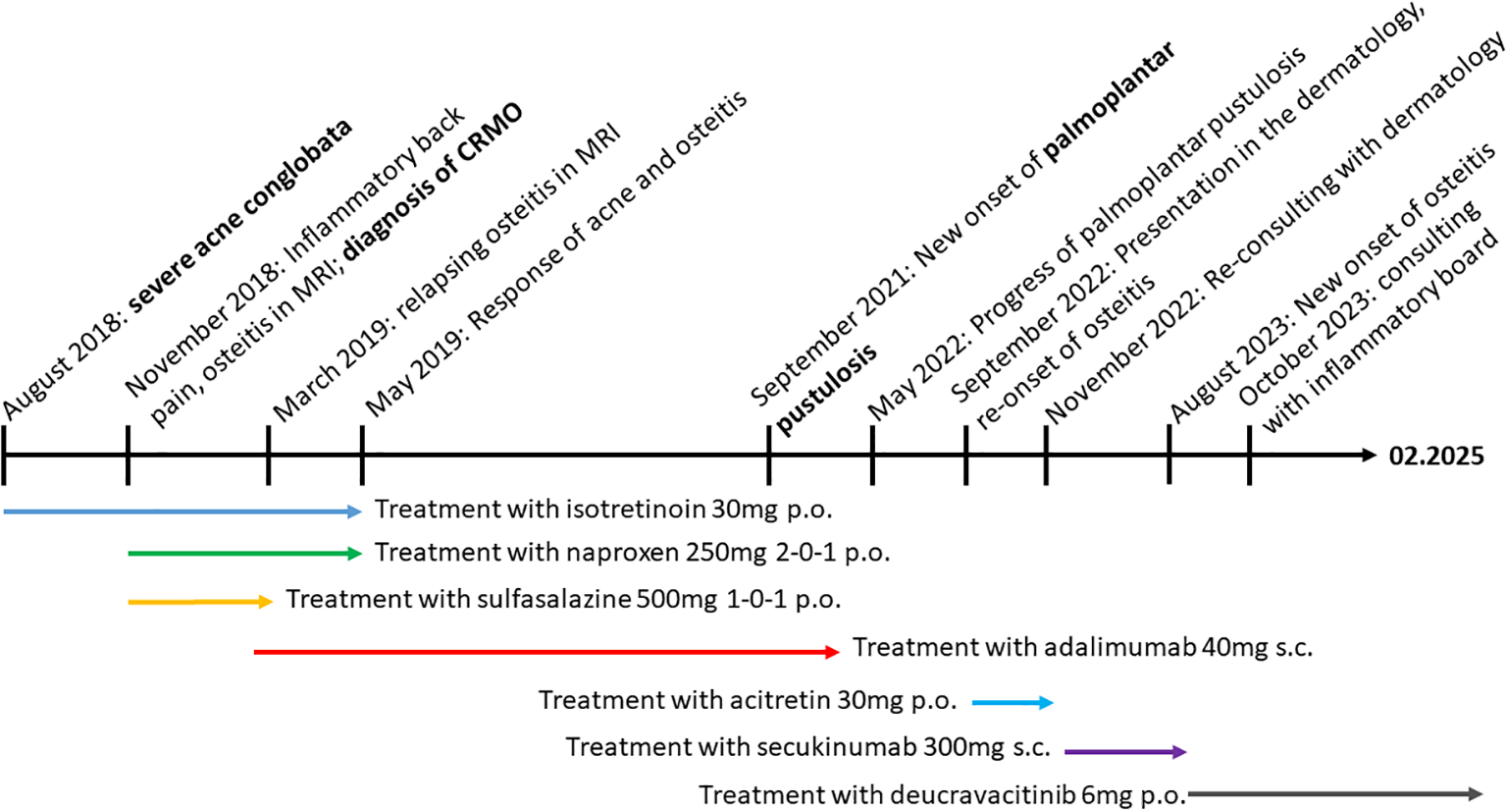
Timeline. Clinical history and therapeutic interventions of the case.

In October 2023, during our interdisciplinary dermatology-rheumatology consultation, we decided to discontinue secukinumab and to initiate treatment with the selective TYK-2 inhibitor deucravacitinib. After four weeks of therapy, the inflammatory pain in the back and lower extremities improved. The skin initially showed a mixed response, with a few relapses of psoriatic lesions (**Figure 2**). Moderate acne-like skin reactions developed on the back, which we attributed to deucravacitinib as a side effect (**Figure 3**). These reactions were treated topically with adapalene and benzoyl peroxide, and systemically with 50 mg doxycycline, which led to healing. The patient’s condition improved steadily with continued treatment. By March 2024, a whole-body MRI as well as a follow-up MRI of the target lesion in the right knee and pelvis confirmed complete and persistent remission of CRMO (**Figures 1**, **4**). After six months of treatment, the skin lesions have healed completely. After 15 months, the patient is currently still in complete remission from CRMO and psoriasis and is satisfied with the treatment and no severe adverse events due to deucravacitinib were reported (**Figure 5**).

## 3 Discussion

In this case report, we present the case of a 22-year-old man with CRMO who additionally developed psoriasis under adalimumab treatment and was successfully treated with deucravacitinib.

CRMO is a rare condition with recurrent osteoarticular inflammation that can be associated with dermatological diseases such as psoriasis, palmoplantar pustulosis and acne (12). There is generally low evidence and currently no established treatment guidelines. Therefore, treatment recommendations have to be primarily deducted from clinical experience, case series and individual case reports like this. Initial therapy typically involves symptomatic and anti-inflammatory treatment with nonsteroidal anti-inflammatory drugs (NSAIDs) (5). This approach can be supplemented with disease-modifying antirheumatic drugs (DMARDs) such as methotrexate, sulfasalazine, and short courses of glucocorticoids (6). In our case, the combination of naproxen and sulfasalazine did not result in the desired remission of CRMO and led to a significant worsening of acne.

Differential diagnosis of chronic inflammatory osteoarticular diseases, particularly those that may share clinical features with CRMO, includes conditions such as synovitis acne pustulosis hyperostosis osteitis (SAPHO) and Pyogenic arthritis, pyoderma gangrenosum, and acne (PAPA) syndrome. SAPHO typically presents with sterile osteitis (13), while PAPA is characterized by acute flares of recurrent sterile arthritis and pyoderma gangrenosum (14).

Given the success of the TNFi adalimumab in treating chronic inflammatory diseases associated with CRMO and its reported effectiveness in CRMO cases, our patient was switched to adalimumab. The goal of this treatment was not only to relieve the inflammatory back pain but also to achieve remission of osteomyelitis to prevent fractures and associated neurological complications in future. We also anticipated that inhibiting TNF-α would improve the patient’s acne conglobata, which did occur. Under adalimumab treatment, our patient experienced good symptom control of CRMO for almost three years. Unfortunately, he then developed a debilitating form of psoriasis, which we suspected being a paradox reaction to TNF-α inhibitors.

Although TNFi is considered an effective second-line treatment, a small proportion of patients develop paradoxical skin reactions such as psoriasis or lupus (15, 16). These reactions are typically characterized by complete resolution upon discontinuation of the drug. This has also been observed in patients with CRMO, where psoriasis resolved after stopping TNFi (17). In our patient, we initially suspected adalimumab-induced paradoxical psoriasis. However, since the psoriasis persisted even after discontinuing adalimumab, we now believe it may be a partial symptom of CRMO. However, confirming this is challenging, as no reliable verification methods are currently available.

Since the patient experienced severe pain and significant limitations due to the worsening of skin symptoms, particularly on the hands and feet, treatment with adalimumab had to be discontinued despite its effectiveness in controlling osteomyelitis. Secukinumab was initiated as an alternative. Although secukinumab is an effective treatment option for psoriasis, the patient showed only a partial response and there was progression of CRMO. In addition, abscesses developed under IL-17i, requiring surgical intervention. Finally, in our interdisciplinary consultation and together with the patient as an act of shared decision making, we decided to administer deucravacitinib. Deucravacitinib, a TYK-2 inhibitor approved in 2023 for the treatment of plaque psoriasis, was considered promising due to case reports of CRMO coexisting with psoriasis, TNFi induced or not, responding to JAK inhibitors (11). Deucravacitinib selectively and allosterically inhibits the regulatory domain of the enzyme TYK-2- and inhibits TYK-2-mediated cytokine signaling of IL-12, IL-23, IL-6, INF-α/β, IL-10, IL-11, and IL-22 (18). In contrast to the previously approved JAKi for psoriasis and rheumatic diseases, the effect of deucravacitinib is largely limited to the immune system and does not additionally affect hematopoiesis, bone formation and lipid metabolism. Due to this selectivity, no increased risk of serious cardiovascular events, deep vein thrombosis and pulmonary embolism had been observed with deucravacitinib in phase 3 clinical trials. Since CRMO and psoriasis share a partially overlapping cytokine profile and TYK-2 is also involved in the signaling of IL-6, IL-23 and IL-11, all involved in promoting osteoclast formation (19), we hypothesized that deucravacitinib could be effective for CRMO.

Our patient demonstrated a good and stable response in both psoriasis and osteomyelitis for 15 months so far and, strikingly, no severe adverse events occurred with deucravacitinib in contrast to the severe infections with the IL-17i before. This suggests that deucravacitinib might be an effective and safe treatment option for patients with CRMO and concomitant psoriasis for the first time. However, the efficacy and safety of this treatment needs to be further evaluated in larger case series or even in clinical trials, including platform trials designed to assess treatments for rare diseases such as CRMO (20).

## Data Availability

The raw data supporting the conclusions of this article will be made available by the authors, without undue reservation.
